# Gender Differences and Influencing Factors in Specialty Choices: Findings From One Medical School in China

**DOI:** 10.3389/fpubh.2021.648612

**Published:** 2021-03-26

**Authors:** Kanhua Yin, Liu Yang, Rui Zhang, Difan Zheng, Michael S. Wilkes, Yanni Lai

**Affiliations:** ^1^Medical Education Office, Fudan University, Shanghai, China; ^2^Office of Dean, University of California, Davis School of Medicine, Sacramento, CA, United States; ^3^Department of Endocrinology and Metabolism, Huashan Hospital, Fudan University, Shanghai, China

**Keywords:** medical education, specialty choices, medical student, gender differences, China

## Abstract

**Background:** Gender plays a significant role in the selection of medical specialty. Few studies have been conducted to explore the impact of gender differences on specialty choosing among Chinese medical students.

**Methods:** The specialty choices of 648 students from six consecutive classes in an 8-year MD program were collected and compared between male and female students. A total of 110 students from one graduating class were surveyed by a questionnaire covering 22 career influencing factors. Each factor has a scale of zero to three (zero = no influence, one = mild influence, two = moderate influence, and three = strong influence).

**Results:** Statistically significant gender differences were observed in 10 out of 16 specialties. Most male students limited their specialty choices to surgery (64%), internal medicine (12%), and orthopedics (12%), compared with a relatively diversified pattern in female students. For male students, the top three influencing factors were personal interest, future job prospects for the chosen specialty, and job opportunity in academic medicine. The strongest influencing factors of females were personal interest, specialty-specific knowledge and skills, and the sense of achievement. The expected salary was ranked among the top 10 influencing factors in male but not in females, while the work-life balance was ranked among the top 10 factors in females but not in males.

**Conclusion:** There is a significant gender difference regarding specialty choices among Chinese medical students. Career coaching is needed to help students in their specialty choosing process.

## Introduction

Around the world, the selection of a medical specialty as a career choice has important implications for both medical students and their healthcare system. Medical students' specialty choice is associated with income, prestige, work hours, and lifestyle. For the healthcare system, the distribution of medical specialties has a profound impact on the ability to deliver health care to high need groups. For students, the process of medical specialty decision-making is influenced by many factors, including the mission of the specific school or program, exposure to influential mentors, cultural background, personal characteristics, and social needs (1–7). Having a better understanding of this process can not only guide the medical educators to provide effective career coaching but also help policymakers to correct the maldistribution of healthcare providers.

Numerous studies conducted in both Western and Eastern societies have demonstrated that gender differences impact medical students' specialty choices and are associated with many factors such as external (e.g., parental) perceptions of specialty characteristics, perceived prestige of the specialty, work-life balance, job-related tasks or responsibilities, and personal interest (3, 8–13). While there is an increasing number of women in medical schools and clinical practice areas worldwide (9, 14), gender inequality still exists in many medical specialties, especially in traditionally male-dominated specialties (15, 16).

China faces both an allopathic physician shortage and a physician maldistribution (both geographic- and specialty-related) (17, 18). In order for medical educators and policymakers to intervene, it is critical to understand medical students' specialty preferences and the driving forces behind specialty selection. However, only a handful of studies have been conducted targeting this topic. For example, by surveying 190 Chinese medical students from three medical schools in Fujian, a province in Southeast China, Liang et al. showed that general surgery and internal medicine are the two most preferred specialties. In contrast, geriatric medicine and psychiatry were the least selected specialties (19). Also, the influencing factors in medical students' specialty choices were evaluated by several prior studies, using both qualitative and quantitative methods (20–22). Personal interest, clinical rotation experience, specialty development prospective, and future income were identified as important decision-making factors. In addition, Chinese medical students' attitudes toward one particular medical specialty or non-traditional medical careers were also evaluated (23, 24). However, none of these studies focuses on the potential gender difference in medical students' specialty choices, and most of these studies were small-scale analyses with <300 students enrolled. In the current study, by analyzing a sizeable cohort of medical students (>600 students), we aimed to examine gender differences in medical students' specialty preferences in a leading Chinese medical school and identify the motivations behind those specialty choices.

## Methods

### Setting and Participants

All medical students from six successive classes (Class 2012–2017) in an 8-year MD program at Fudan University, China, were involved in this study. All students in this premier program had excellent academic credentials upon admission to medical school (National College Entrance Exam scores were ranked at top 0.1–1%). The curriculum consisted of a 2-years of general education followed by 4 years of combined basic medical science and clinical knowledge training. Students selected their career specialty at the end of their 6th year. Students then continued their training for two additional years, which included combined clinical and research experiences in their own specialties. As such, the training was a “2 + 4 + 2” model. Specialty choice at the end of the 6th year represented the students' residency selection after graduation.

### Data Collection and Survey

All students' specialty choice and gender were collected from an internal database at the Medical Education Office of Fudan University. Specialties were categorized into 16 categories according to the Chinese National Standard of Subject Classification (GBT 13745-2009): internal medicine, surgery, obstetrics and gynecology (OB/GYN), pediatrics, family medicine, neurology, orthopedics, anesthesiology, diagnostic radiology, therapeutic radiology, dermatology, ophthalmology, otolaryngology, pathology, rehabilitation medicine, and psychiatry. Of note, three oncology branches—medical, surgical, and gynecologic oncology—were classified into internal medicine, surgery, and OB/GYN, respectively.

A 34-item questionnaire was delivered to all final year students (Class of 2017) to explore factors that might have influenced their specialty choice. Using a structured focus group of 10 final-year medical students, we derived a total of 22 factors to include in the questionnaire. Students were asked to rate influencing factors: zero = no influence, one = mild influence, two = moderate influence, and three = strong influence. A factor with a mean score over 2.5 was considered a strong influence, while a score between 2.0 and 2.5 was considered a moderate influence.

### Statistical Analysis

Continuous data were expressed as mean ± standard deviation. Categorical data were compared by the Chi-square test as long as the observed cell counts were over five. Otherwise, Fisher's exact test was used. A *p* < 0.05 was considered statistically significant. All statistical analysis was performed using SPSS 19.0 (Chicago, IL, USA).

## Results

### Specialty Choices and Gender Differences

The specialty choices of a total of 648 students (mean age 23.9 ± 1.3 years, 283 males and 365 females) from six consecutive classes were collected and are summarized in Table 1. The number of students per class varied from 97 to 113, and the mean sex ratio for each class was 0.78 ± 0.14 (range: 0.59–0.98).

**Table 1:** Specialty choices of 648 Chinese medical students.

	**Male (*****n*** **= 283)**		**Female (*****n*** **= 365)**		
**Specialty**	**Number**	**%**	**Number**	**%**	***P-*value**
Surgery (*n* = 230)	180	63.60	50	13.70	<0.001*
Internal medicine (*n* = 164)	35	12.37	129	35.34	<0.001*
Neurology (*n* = 39)	11	3.89	28	7.67	0.045*
Ophthalmology (*n* = 37)	4	1.41	33	9.04	<0.001*
Orthopedics (*n* = 36)	33	11.66	3	0.82	<0.001*
OB/GYN (*n* = 34)	3	1.06	31	8.49	<0.001*
Dermatology (*n* = 24)	2	0.71	22	6.03	<0.001*
Therapeutic radiology (*n* = 21)	2	0.71	19	5.21	0.001*
Diagnostic radiology (*n* = 19)	7	2.47	12	3.29	0.542
Anesthesiology (*n* = 18)	2	0.71	16	4.38	0.006*
Pediatrics (*n* = 10)	0	0.00	10	2.74	0.003*
ENT (*n* = 7)	4	1.41	3	0.82	0.705
Pathology (*n* = 4)	0	0.00	4	1.10	0.136
Rehabilitation medicine (*n* = 3)	0	0.00	3	0.82	0.261
Psychiatry (*n* = 2)	0	0.00	2	0.55	0.507
Family medicine (*n* = 0)	0	0.00	0	0.00	–

*Asterisk indicates statistical significance*.

**Figure 1:**
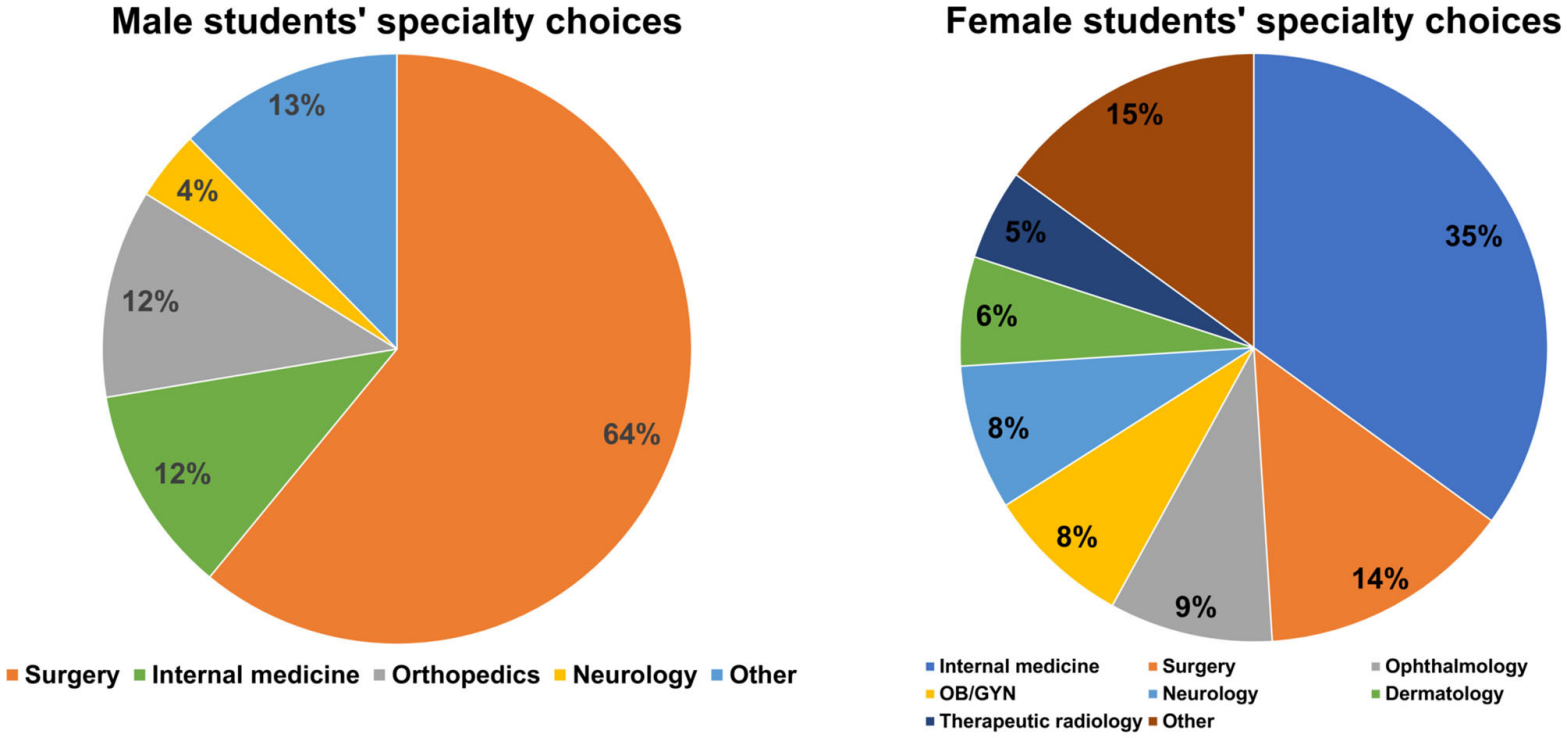
The male and female medical students' specialty choices.

Eighty-eight percent of male students focused their choices only on three specialties: surgery (*n* = 180, 63.6%), internal medicine (*n* = 35, 12.3%), and orthopedics (*n* = 33, 11.7%). In contrast, female students' specialty choices were more diverse (Figure 1). The most often selected specialties included 35% internal medicine (*n* = 129), 14% surgery (*n* = 50), 9% ophthalmology (*n* = 33), and 9% OB/GYN (*n* = 31).

Statistically significant gender differences were observed in 10 out of 16 specialties (62.5%): A significantly higher proportion of male students chose surgery and orthopedics (both *p* < 0.001); more female students chose internal medicine (*p* < 0.001), neurology (*p* = 0.045), ophthalmology (*p* < 0.001), OB/GYN (*p* < 0.001), dermatology (*p* < 0.001), therapeutic radiology (*p* < 0.001), anesthesiology (*p* = 0.006), and pediatrics (*p* = 0.003).

Six specialties [pediatrics (*n* = 10), ENT (*n* = 7), pathology (*n* = 4), rehabilitation (*n* = 3), psychiatry (*n* = 2) and family medicine (*n* = 0)] were selected by fewer than 10 students from the entire 6-year cohort. Except for ENT (where four students were male), all students selecting these less popular specialties were female.

### Influencing Factors

One hundred and ten students out of 113 medical students (97%) from the Class of 2017 completed the questionnaire. This included 60 female and 50 male students. The top 10 influencing factors of both male and female students are summarized in Table 2. The mean scores of all these factors were above two (i.e., moderate influence).

**Table 2:** Top 10 influencing factors in specialty choices.

**Rank**	**Male**	**Mean score**	**Female**	**Mean score**
1	Personal interest	2.6	Personal interest	2.7
2	Future job prospects	2.6	Specialty-specific knowledge and skills	2.6
3	Opportunity in academic medicine	2.5	Sense of achievement	2.5
4	Expected salary	2.5	Prestige/reputation of clinical supervisor	2.5
5	Prestige/reputation of clinical supervisor	2.5	Opportunity in academic medicine	2.4
6	Specialty is technically demanding	2.4	Future job prospect	2.4
7	Specialty-specific knowledge and skills	2.4	Working environment	2.3
8	Working environment	2.4	Job opportunity in general	2.2
9	Sense of achievement	2.3	Work-life balance	2.2
10	Job opportunity in general	2.2	Specialty is technically demanding	2.2

For male students, personal interest (*x* = 2.6), future job prospects (*x* = 2.6), and opportunities in academic medicine (*x* = 2.5) were the three highest ranked influencing factors. In contrast, for female students, personal interest (*x* = 2.7), specialty-specific knowledge and skills (*x* = 2.6), sense of achievement (*x* = 2.5), and prestige or reputation of their clinical supervisor (*x* = 2.5) were the four strongest influencing factors.

Males ranked expected salary (*x* = 2.5) the fourth most important criterion, but this was not the top 10 factors for females. In contrast, work-life balance (*x* = 2.2) was ranked in the top 10 factors for female students (No. 9) but was not highly ranked in males.

## Discussion

This study is the first report of Chinese medical students' specialty preferences and selection factors analyzed by a gender stratification to the best of our knowledge. There are significant gender differences in specialty selection preferences among Chinese medical students. Female students had more diverse specialty choices compared with male students. Male students placed more weight on career and job prospects, salary, and technical challenges than females, while female students were more concerned about work-life balance and the sense of achievement.

Fifty-six percent of the study group were female students, which is comparable to the overall Chinese medical student population and is consistent with the global phenomenon that more females are entering medical schools and medical practice worldwide, often referred to as the “feminization” of medicine (9, 25). However, gender inequality still exists widely in both medical schools and hospitals. For example, females still represent a small minority in many surgical fields. Therefore, there are few female surgeons available to serve as role models (15). In contrast, male faculty are often a minority in fields like OB/GYN making finding male role models difficult for students (26).

In our study, statistically significant gender differences were observed in ten specialties (63%), suggesting that male and female Chinese medical students make very different specialty choices. Surgery and orthopedics were heavily male-dominated choices, while female students' selection is more diverse and gravitates toward internal medicine, ophthalmology, neurology, dermatology, radiology, anesthesiology, pathology, OB/GYN, and pediatrics. This is consistent with studies from other countries showing similar trends whereby females select OB/GYN, pediatrics, and primary care, while males select more procedure-oriented specialties (2, 5, 8–10, 13).

Understanding specialty choice is complex and multifactorial with influencing factors from internal (student-related personal factors) and external factors (family, peers, role models, potential earning power, etc.). External factors that are perceived to be “negative” such as an unfriendly working environment, scarcity of female role models, and a lack of career guidance and coaching may drive students away from certain careers. Given the heavy clinical workload required in Chinese medical practice and the extremely short time devoted to the doctor-patient relationship, career choices that devalue work-life balance, a disregard for personal wellness, and specialties that allow little time for family life seem less attractive to female medical students. Traditionally, female medical students have been exposed to few female role models in specialties like surgery. Those role models that do exist may have adopted traditional male values and work-life patterns. Without career consulting and coaching, male and female medical students often select stereotype roles and are not aware of other potentially more attractive specialties. It would be worth exploring the impact of career counselors or career advisors on specialty selection (27). Male students tend to select specialty based on the male's traditional role in Chinese society as head of household, main income earner, and a competitor. This may help explain why many female students chose the less prestigious specialties (i.e., specialties chosen by <10 students). It is important to point out that students enrolled in this study were the products of China's “one-child policy.” High family expectations, especially expectations placed on male students, may contribute to the current gender distribution.

It should be noted that only 10 students (all females) chose pediatrics, two students chose psychiatry, and no student chose family medicine over six consecutive years. However, an estimated shortage of 200,000 pediatricians' poses is a major threat to Chinese children (28). Similarly, there are only 1.2 family physicians per 10,000 population, far fewer than that in other developed countries (29). There are also significant shortages in licensed psychiatrists and allied mental health professionals in China (30). Students wrote in their surveys that long working hours, a high chance of getting involved in the doctor-patient physical conflict (mainly with patients' parents), and a relatively low salary are three main reasons that make pediatrics and psychiatry less attractive.

This study has some limitations. The results were based on a single medical school located in a large metropolitan region. The results may not represent all Chinese medical students, especially those in remote areas of the country. Moreover, as the study design is cross-sectional, it is impossible to demonstrate the causality between influencing factors and gender. Establishing and analyzing a multi-school, multi-year, longitudinal cohort covering different geographical areas and various types of degree programs may better elucidate the impact of gender on Chinese medical students' specialty choices.

## Conclusions

There are significant gender differences in specialty preferences among Chinese medical students. Male students tend to hold more practical and utilitarian attitudes toward their future specialty choices, while female students are more concerned about work-life balance and sense of achievement.

## Data Availability Statement

The raw data are available on reasonable request due to privacy or ethical restrictions.

## Ethics Statement

The studies involving human participants were reviewed and approved by The Institutional Review Board of Fudan University Shanghai Medical College. The patients/participants provided their written informed consent to participate in this study.

## Author Contributions

KY, LY, MSW, and YL were involved in the conceptualization and design of this study. KY, LY, and DZ collected the data. KY, RZ, and DZ analyzed the data and interpreted the results. KY and RZ drafted the initial manuscript with critical feedback from MSW and YL. All authors read and approved the final manuscript.

## Conflict of Interest

The authors declare that the research was conducted in the absence of any commercial or financial relationships that could be construed as a potential conflict of interest.

## Abbreviations

ENT: Eye, nose, and throat (aka: otolaryngology)
GBT: Guobiao Standard (aka: Chinese National Standard)
OB/GYN: obstetrics and gynecology.
